# High-Fat Diet Promotes Macrophage-Mediated Hepatic Inflammation and Aggravates Diethylnitrosamine-Induced Hepatocarcinogenesis in Mice

**DOI:** 10.3389/fnut.2020.585306

**Published:** 2020-11-16

**Authors:** Huiying Fu, Biqiang Tang, Jiali Lang, Yueguang Du, Beibei Cao, Lushuai Jin, Mingsun Fang, Zhiming Hu, Changpei Cheng, Xia Liu, Qiyang Shou

**Affiliations:** ^1^The Second Clinical Medical College & Second Affiliated Hospital, Zhejiang Chinese Medical University, Hangzhou, China; ^2^Basic Medicine College, Zhejiang Chinese Medical University, Hangzhou, China; ^3^Department of Hepatobiliary and Pancreatic Surgery, Zhejiang Provincial People's Hospital, Hangzhou, China; ^4^First Clinical Medical College, Guizhou University of Traditional Chinese Medicine, Guiyang, China

**Keywords:** liver cancer, tumorigenesis, collagen, inflammatory factors, fatty acid

## Abstract

It has been reported that diet and nutrition play important roles in the occurrence and development of hepatocellular carcinoma (HCC). In this study, we investigated the potential tumor-promoting mechanisms of a high-fat diet (HFD) in mice with dietondiethylnitrosamine (DEN)-induced hepatocarcinogenesis. HFD significantly decreased the survival rate and induced severe liver dysfunction in DEN-induced mice, as indicated by increased serum glutamic-pyruvic transaminase (ALT), glutamic oxalacetic transaminase (AST), and alkaline phosphatase (ALP) levels and increased liver index, liver nodule count, and γ-glutamyltransferase (γ-GT) activity. Moreover, an increased number of fat droplets and HCCs were found in the livers of the HFD mice, who displayed little collagen in and around the liver cancer groove and the infiltration of large number of inflammatory cells, such as macrophages, compared with the control mice. HFD also significantly increased proliferating cell nuclear antigen (PCNA), nuclear factor-κB (NF-κB), cyclin D1, tumor necrosis factor (TNF), and interleukin-1 (IL-1) expression levels in the liver. *In vitro*, we found that the inducible nitric oxide synthase (iNOS) percentage increased in macrophages after palmitic acid treatment, as well as the secretion of inflammatory factors and cytokines such as interleukin-6(IL-6), interleukin-10(IL-10), CCL2, Interferon γ (IFN-γ), and TNF. Thus, our results demonstrate that an HFD may promote DEN-induced hepatocarcinogenesis in mice by destroying liver function and enhancing the inflammatory response by recruiting and polarizing macrophages in the liver. This study could therefore provide new insights into the tumor promoting effects of an HFD in HCC.

## Introduction

Epidemiological studies have revealed that the incidence of many types of cancer has declined with the development of science and technology during the last 10 years; however, the incidence and mortality of liver cancer have risen sharply (1). This may partially be due to high rates of non-alcoholic fatty liver disease (NAFLD), obesity, and diabetes, since NAFLD, obesity, and metabolic syndromes account for 30–40% of the increase in hepatocellular carcinoma (HCC) incidence in developed countries (2). Patients with NAFLD have a 7-fold higher risk of liver cancer than the general population and 80% of patients with cirrhosis and NAFLD develop HCC (3). The development of NAFLD into cryptogenic cirrhosis, or even HCC, is a long and dangerous process whose carcinogenic mechanism is currently poorly understood.

Diet is the key strategies for improving NAFLD, and it is already known to play a key role in controlling diseases such as diabetes and high blood pressure and a growing body of evidence suggests that it can also help treat cancer (4). Therefore, it is crucially important to study the effect of diet on the occurrence of liver cancer in order to prevent and treat the disease. High-fat diets (HFDs) are known to be harmful and many studies have linked them to a variety of cancers, including gastrointestinal cancers. A recent study found that an HFD promotes the growth of colorectal cancer by disrupting the balance between bile acids in the gut and triggering hormonal signals that could allow cancer cells to thrive (5). Moreover, an HFD has also been shown to alter the intestinal microbial composition of mice, leading to reduced immune defenses against bowel cancer and thus increasing the incidence of cancer (6). An HFD may also increase the risk of breast cancer by causing changes in the breast (e.g., excessive cell growth) and immune cells that can eventually lead to precancerous changes and rapid progression to breast cancer (7). Fatty liver disease, also known as NAFLD, is mainly caused by excessive fat and sugar intake combined with a sedentary or inactive lifestyle. If NAFLD becomes chronic it can lead to nonalcoholic steatohepatitis (NASH), both of which are the most common causes of liver cancer or HCC, along with chronic viral infections.

Scientists believe that metabolic imbalance can cause immune cells to activate and migrate to the liver where they interact with liver cells to trigger an inflammatory response that damages liver tissue and drives liver disease (8). However, the mechanism that causes diseases such as NAFLD, steatohepatitis, and liver cancer remain poorly understood. Here, we observed the effects of an HFD in a chemically-induced liver cancer model and investigated the possible mechanisms by which an HFD promotes liver cancer.

## Materials and Methods

### Animals and Diets

Thirty-five male C3h mice were purchased from Beijing Life River Laboratory Animal Technology (Beijing, China) and randomly divided into a control group (*n* = 9), a DEN group (*n* = 13), and a DEN+HF group (*n* = 13). The control group drank fresh, sterile tap water for 22 weeks, while the others drank water containing 30 mg/mL of dietondiethylnitrosamine (DEN) solution (1, 2). The control and DEN-only groups were fed regular forage, whereas the DEN+HF group was fed high fat forage containing 5% shortening, 5% lard, and 1% cholesterol The growth and food intake of each mouse was monitored closely and their body weight was measured once a week. At the end of experiments, the mice were sacrificed by euthanasia using an overdose of CO_2_ in a closed plastic chamber. The liver was removed and photoed for the nodules immediately after death. Then the livers were weighted and calculated the hepatic index = the weight of liver (g)/mice body weight (kg). The cancer incidence was calculated as the number of animals with at least one nodule. Cancer incidence (%) = the number of mice with nodule/the total number mice for each group ×100%. Then the right lobe fixed with 10% formaldehyde solution for histological examination. The remaining liver tissue was frozen rapidly with liquid nitrogen and stored at −80°C until further analysis. All experiments were conducted according to the Animal Health Care and Use Guidelines of the Zhejiang Chinese Medical University.

### Biochemical Analysis

Serum alanine aminotransferase (ALT), aspartate aminotransferase (AST), and alkaline phosphatase (ALP) levels were determined using a Hitachi 7020 automatic biochemical analyzer (Tokyo, Japan) and the corresponding kit (Shengnong/Desai Diagnostic Technology, Shanghai, China). To measure γ-glutamyltransferase (γ-GT) content, 10% liver homogenates were centrifuged at 2,000 rpm for 10 min and the supernatant analyzed using a Varioscan Flash Multifunctional Enzyme Labeling Instrument (Finland Thermo Fisher) with γ-GT and total protein (TP) kits from the Nanjing Jiancheng Biology Engineering Research Institute (Nanjing, China). γ-GT levels were calculated as γ-GT per gram of protein in the liver (U/g prot).

### Histology

Liver tissues were dissected, embedded in paraffin, cut into 4 μm sections, and stained with hematoxylin and eosin (H&E) and Masson's trichrome. An Eclipse 80i microscope (Nikon, Tokyo, Japan) was used to image the sections at 400× magnification. Five non-overlapping Masson's tricolor images were taken for each section and analyzed with Image Pro Plus 5.0 software.

### Immunohistochemistry

Collagen 1, collagen 3, proliferating cell nuclear antigen (PCNA), nuclear factor-κB (NF-κB), tumor necrosis factor α (TNF-α), and interleukin-1β (IL-1β) expression were detected in the liver tissue by immunohistochemistry. Briefly, tissue sections were dewaxed, water was added, and heat-induced antigens were extracted for 60 min. The sections were then incubated with 3% H_2_O_2_ solution to block peroxidase activity for 10 min at 37°C with anti-collagen 1 (1:100), anti-collagen 3 (1:100), anti-NF-κB (1:100), anti-IL-1β (1:100), anti-PCNA-1 (1:100), and anti-TNF (1:100) antibodies. The sections were washed three times in phosphate buffered saline (PBS), incubated for 45 min at 37°C with secondary antibodies (1:100), and washed with PBS a further three times for 3 min each. According to the manufacturer's instructions, 3,3′-diaminobenzidine tetrahydrochloride (DAB) immunohistochemical staining was performed and an Eclipse 80i microscope was used to image the sections at 400× magnification. Six non-overlapping visual fields were randomly imaged and analyzed using Image Pro Plus 5.0 software.

### RNA Extraction and mRNA Expression Analysis by Quantitative PCR (qPCR)

Total RNA was extracted from the liver tissue samples and reverse transcribed into cDNA using Triazole reagent and reverse transcriptase reagent (Bao Bioengineering, Dalian, China) according to the manufacturer's instructions. The relative mRNA expression of the target genes and GAPDH was determined by qPCR on an MJOpticon system using iTaq Universal SYBR Green Hypermix (Bao Bioengineering) and the following forward and reverse primers: PCNA F-5′-AGCATGGACTCGTCTCACG and R-5′-GCGCAGAGTAAGCTGTACCAA; NF-κB F-5′-ATGGCAGACGATGATCCCTAC and R-5′-CGGAATCGAAATCCCCTCTGTT; Cyclin D1 F-5′-GCGTACCCTGACACCAATCTC and R-5′-ACTTGAAGTAAGATACGGAGGGC; GAPDH F-5′-AGGTCGGTGTGAACGGATTTG and R-5′- GGGGTCGTTGATGGCAACA. Relative mRNA expression was normalized to GAPDH and calculated using the 2^−ΔΔ*Ct*^ method.

### Flow Cytometry

After palmitic acid (PA; Sigma-Aldrich, Wuxi, China) treatment, cells were harvested, washed with cold PBS, and fixed in ice-cold 70% ethanol overnight at −20°C. The fixed cells were incubated with iNOS antibodies for 30 min at 4°C in the dark and analyzed (>1 × 10^4^ cells) using a BD FACSCanto Plus instrument with BD FACSDiva software.

### Cytometric Bead Array (CBA)

A CBA Flex Set kit (BD Biosciences, San Jose, CA, USA) was used to assess IL-6, IL-10, interferon gamma (IFN-γ), TNF-α, and C-C motif chemokine ligand 2 (CCL2) levels in the cell culture supernatant according to the manufacturer's instructions. Data were analyzed using CellQuest software (BD Biosciences) and BD Pharmingen (BD Biosciences).

### Murine Peritoneal Macrophages

Murine peritoneal macrophage cells were isolated from adult male C3h mice following the previous reported protocol (3). Briefly, 1 ml of 3% Brewer thioglycolate medium were injected into the mice peritoneal cavity and allow inflammatory response to proceed for 4 days. The macrophages were collected cultured with DMEM medium supplemented with 10% FBS. Then the cells were planted in a 24-well plate at a density of 1 × 10^6^ cells/well. After 24 h cultured, the cell were treated with almitic acid (PA, Sigma-Aldrich, US) at the dose of 100 and 200 μM for 48 h. Then the medium was collected for CBA assay, and the cells were collected for FACS or Immunofluorescence.

### Immunofluorescence

The cell were fixed with methanol (−20°C stock) for 10 min and rinsed with PBS (0.01 M; pH 7.4) three times. The cells were then incubated in 0.2% Triton X-100 for 30 min, blocking solution (5% NGS in PBST) for 1 h, and P65 antibodies (1:100) at 25°C for 2 h. The cells were rinsed with PBS three times, incubated with secondary antibodies at 25°C for 1 h, rinsed with PBS three times, incubated with DAPI at 25°C for 5 min, and rinsed with PBS a further three times.

### Statistical Analysis

All statistical analyses were conducted using SPSS V.170 (SPSS, Chicago, IL). Data are expressed as the mean ± SEM. The survival rate was evaluated using the Kaplan–Meier analysis. All the differences between groups were evaluated by one-way analysis of variance (ANOVA) using the statistical package for the social sciences (SPSS) 18.0 software (SPSS Inc., Chicago, IL, USA) and *P*-values of < 0.05 were considered statistically significant.

## Results

### HFD Increases Mortality and Liver Dysfunction in a DEN-HCC Model

**Figure 1:**
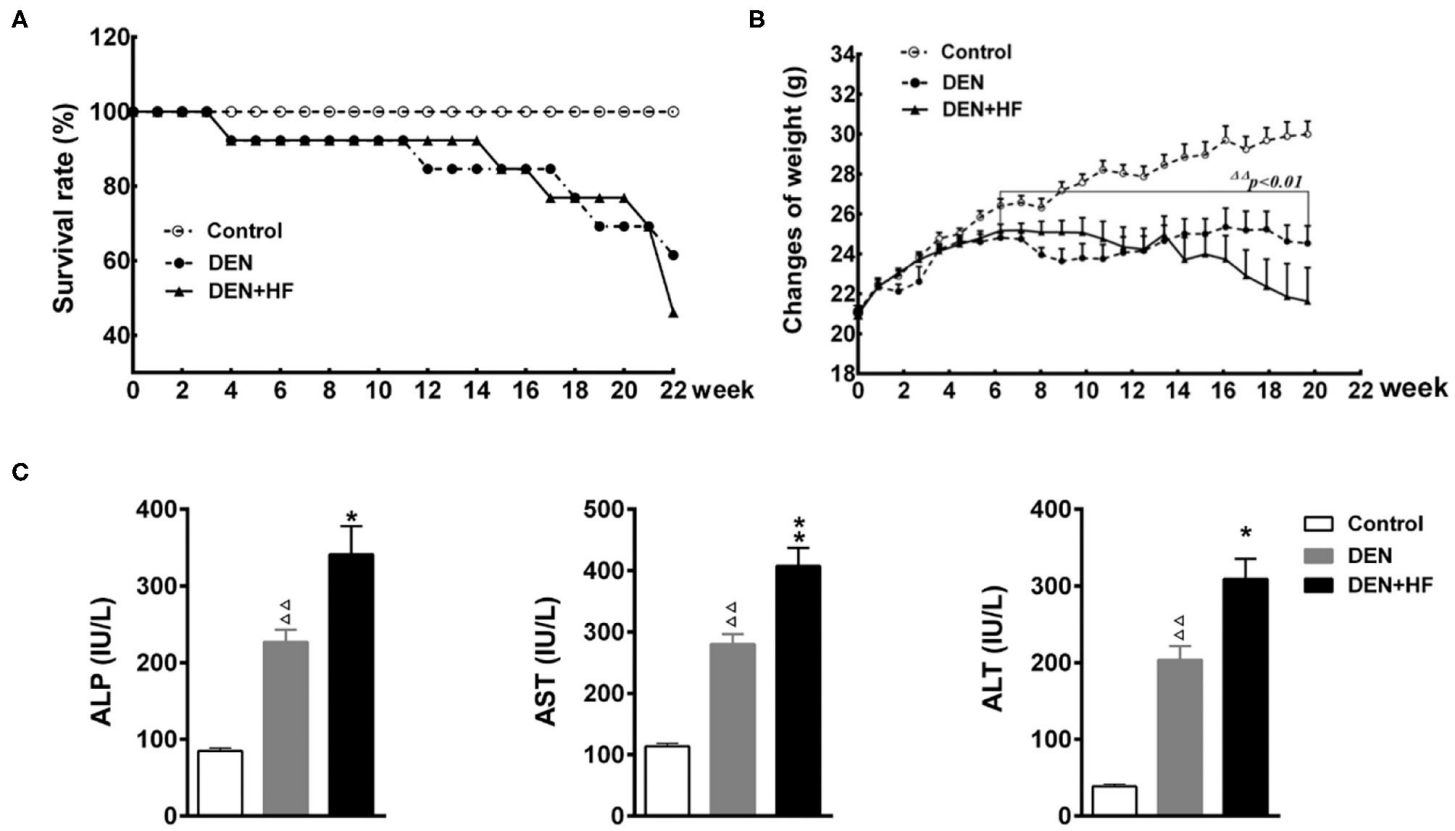
Effect of a high-fat diet on the survival, weight, and plasma liver enzyme levels in a mouse model of hepatocellular carcinoma. **(A)** Kaplan–Meier survival curve of mice during 22 weeks; **(B)** body weight was measured for 22 weeks. **(C)** Plasma alanine aminotransferase (ALT), alkaline phosphatase (ALP), and aspartate aminotransferase (AST) levels. DEN, dietondiethylnitrosamine (DEN)-fed group; DEN + HF, DEN and high-fat diet (HFD)-fed group. ^ΔΔ^*P* < 0.01 vs. control, *P < 0.05 and **P < 0.01 vs. DEN only.

The survival rate of all three groups of mice was assessed for 22 weeks. The survival rate of the DEN and DEN+HF groups began to decrease at week 4, dropping to 61.54 and 46.15%, respectively, by week 22 (Figure 1A). Moreover, the weight of the DEN and DEN + HF mice significantly decreased between weeks 7 and 22, with no difference in weight change found between the DEN and DEN + HF groups (Figure 1B). Since the death of the DEN mice could have been due to severe liver injury and atrophy leading to liver failure, we investigated their liver function. As shown in Figure 1C, serum ALT, ALP, and AST levels were significantly higher in the DEN and DEN + HF mice, with the DEN+HF mice displaying the greatest increase.

### HFD Increases the Incidence of Liver Cancer

**Figure 2:**
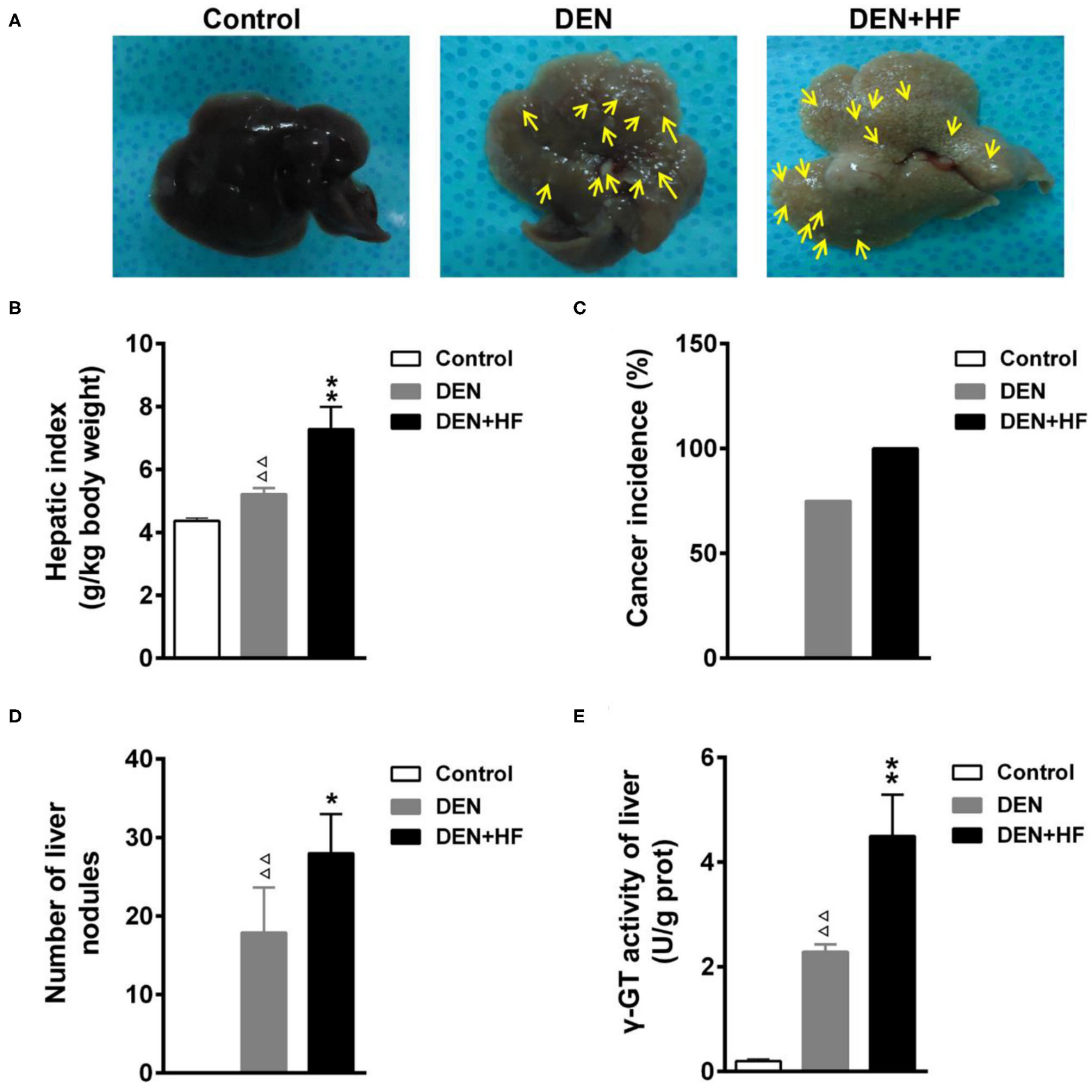
Effect of a high-fat diet on tumorigenesis in a mouse model of hepatocellular carcinoma. **(A)** Representative livers from each group, round gray nodules were indicated by the yellow arrows. **(B)** Liver index was calculated as the wet liver weight to body weight ratio. **(C)** Cancer incidence was calculated as the number of animals with at least one nodule. **(D)** Average number of tumors in each group. **(E)** γ-GT activity in the liver as a marker of premalignant and malignant lesions. DEN, dietondiethylnitrosamine (DEN)-fed group; DEN + HF, DEN and high-fat diet (HFD)-fed group. ^ΔΔ^*P* < 0.01 vs. control; *P < 0.05 and **P < 0.01 vs. DEN only.

We investigated the potential effect of an HFD on tumorigenesis. As shown in Figure 2, tumor incidence in the DEN+HF and DEN groups was 100 and 75%, respectively, whereas no tumors were found in the control group. Round gray nodules indicated by the yellow arrows, were observed on the surface of livers from the DEN-treated mice when examined by the naked eye (Figures 2A,C). In comparison, the livers of the DEN + HF mice were yellow in color and displayed more gray and white round nodules on their surface. Moreover, liver index, liver nodule number, and γ-GT activity were significantly higher in the DEN group than the control group (*P* < 0.01; Figures 2B,D,E).

### HFD Enhances Histopathological HCC Changes

**Figure 3:**
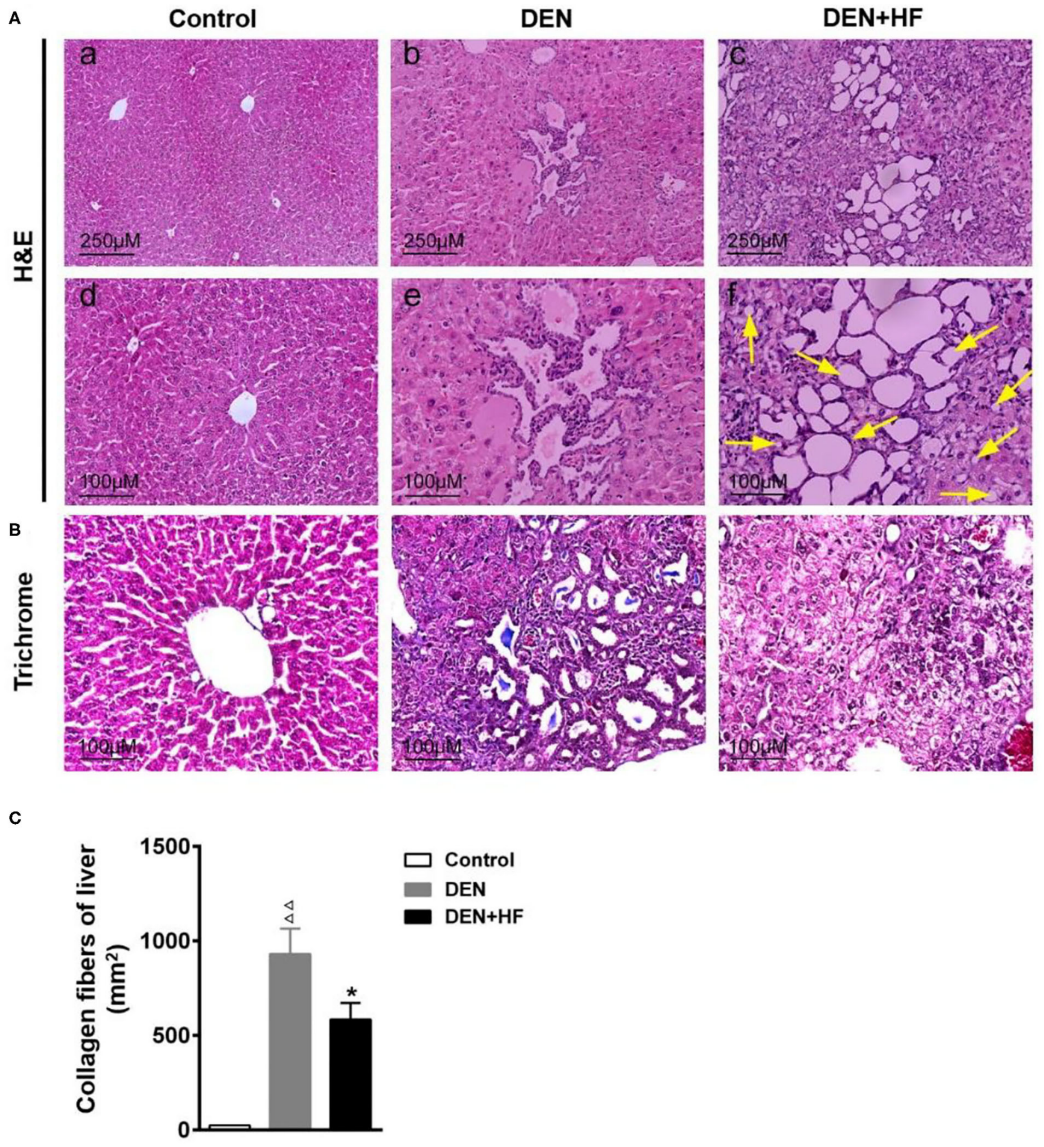
Histopathological examination of liver tissue. **(A)** H&E staining and light microscopy of liver histopathology, yellow arrow indicated the micro- and macrovesicular steatosis. Scale bars: 250 μm (upper), 100 μm (lower). **(B)** Collagen accumulation was visualized by Masson trichrome staining. Scale bar: 100 μm. **(C)** Liver collagen deposition was quantified by measuring the trichrome-positive (blue) area. DEN, dietondiethylnitrosamine (DEN)-fed group; DEN + HF, DEN and high-fat diet (HFD)-fed group. ^ΔΔ^*P* < 0.01 vs. control; *P < 0.05 vs. DEN only.

H&E staining of the liver sections revealed nests of differentiated hepatoma cells which were identified as HCC and mainly existed as stands and trabeculae with bleeding and necrosis in the DEN group (Figure 3A-b,e). More nests were observed microscopically in the DEN + HF group than in the DEN group. Moreover, mixed micro- and macrovesicular steatosis (indicated by yellow arrows) were observed in the liver cells of the DEN + HF group but not in the DEN group (Figure 3A-c,f), whilst Masson staining showed that the liver tissue of the DEN + HF mice had significantly less collagen deposition that that of the DEN mice (Figures 3B,C).

### HFD Promotes Hepatocyte Proliferation and Inflammation

**Figure 4:**
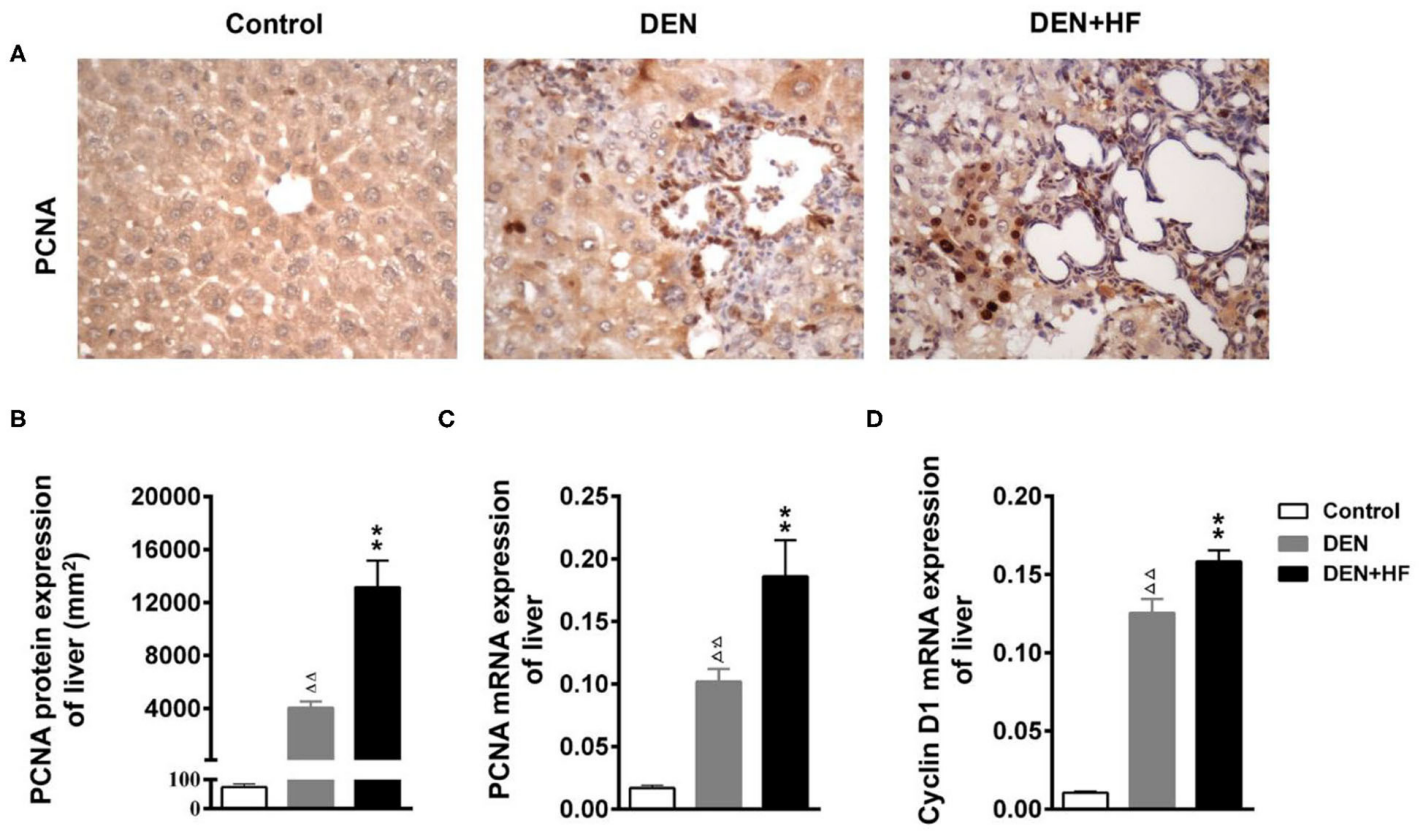
Effect of a high-fat diet on hepatocyte proliferation in the liver of a mouse model of hepatocellular carcinoma. **(A)** Representative images of PCNA expression in liver tissue sections. Scale bar: 100 μm. **(B)** PCNA staining region in immunoreactive hepatocytes expressed as the mean ± SEM. **(C)** PCNA mRNA expression relative to GAPDH was detected by qPCR. Data represent the mean ± SEM (*n* = 9–13). ^ΔΔ^*P* < 0.01 vs. control; **P < 0.01 vs. DEN only. **(D)** Cyclin D1 mRNA expression relative to GAPDH was detected by qPCR. Data represent the mean ± SEM (*n* = 9–13). ^ΔΔ^*P* < 0.01 vs. control, **P < 0.01 vs. DEN only. DEN, dietondiethylnitrosamine (DEN)-fed group; DEN + HF, DEN and high-fat diet (HFD)-fed group.

PCNA is an indicator of cell proliferation that is closely related to DNA synthesis and plays an important role in the initiation of cell proliferation. As shown in Figures 4A–C, PCNA protein and mRNA levels were significantly higher in the DEN and DEN + HF groups than the control group, with DEN + HF mice showing the greatest increase. Moreover, CyclinD1 mRNA levels were also significantly higher in the DEN+HF mice than the DEN mice (Figure 4D).

**Figure 5:**
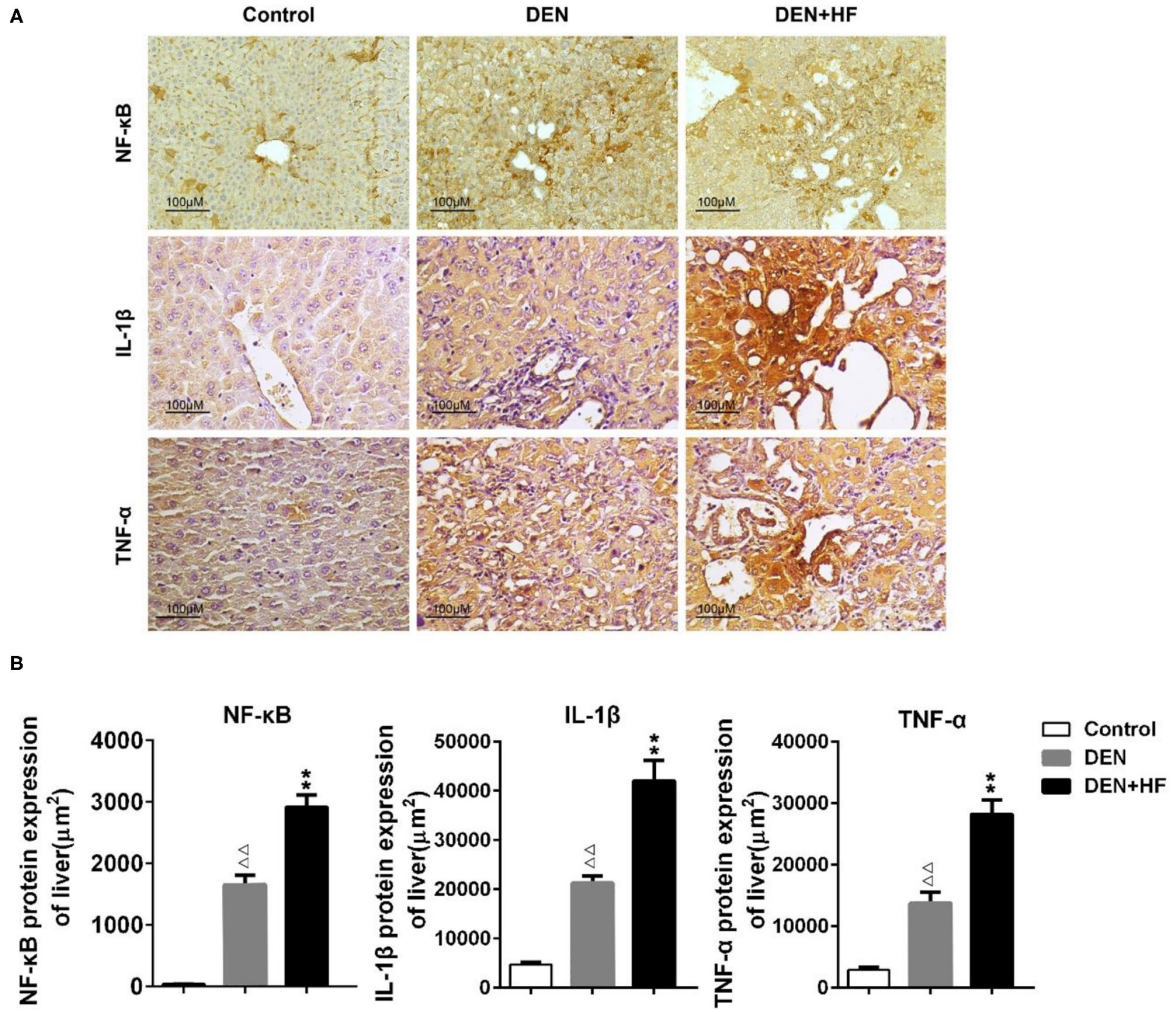
The levels of liver inflammation proteins were detected by immunohistochemistry. **(A)** Representative images of NF-κB, TNF-αand IL-1β expression in liver tissue sections. Scale bar: 100 μm. **(B)** NF-κB, TNF-α, and IL-1β staining region in immunoreactive hepatocytes expressed as the mean ± SEM. ^ΔΔ^*p* < 0.01 vs. control; **p < 0.01 vs. DEN only. DEN, dietondiethylnitrosamine (DEN)-fed group; DEN + HF, DEN and high-fat diet (HFD)-fed group.

**Figure 6:**
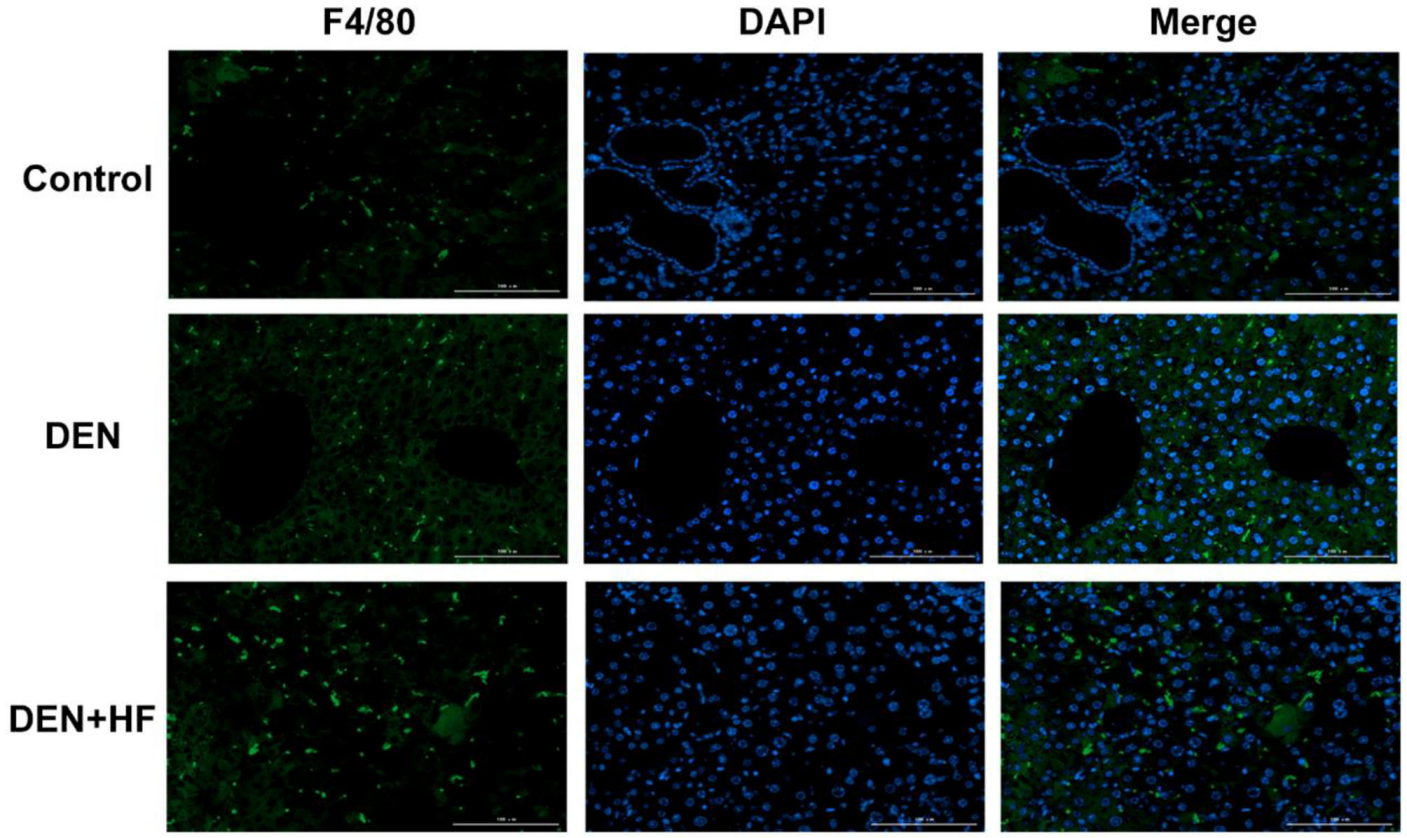
Infiltration of liver macrophages detected by immunofluorescence. Scale bar: 100 μm. DEN, dietondiethylnitrosamine (DEN)-fed group; DEN + HF, DEN and high-fat diet (HFD)-fed group.

We also analyzed the expression of inflammation-associated genes. NF-κB, IL-1β, and TNF-α levels were all significantly increased in the livers of the DEN and DEN + HF mice, with DEN + HF mice showing the greatest increase (Figure 5). Furthermore, macrophage infiltration was found to be significantly higher in the DEN + HF group than in the DEN group (Figure 6).

### PA Treatment Enhances the Secretion of Inflammatory Factors by Macrophages

**Figure 7:**
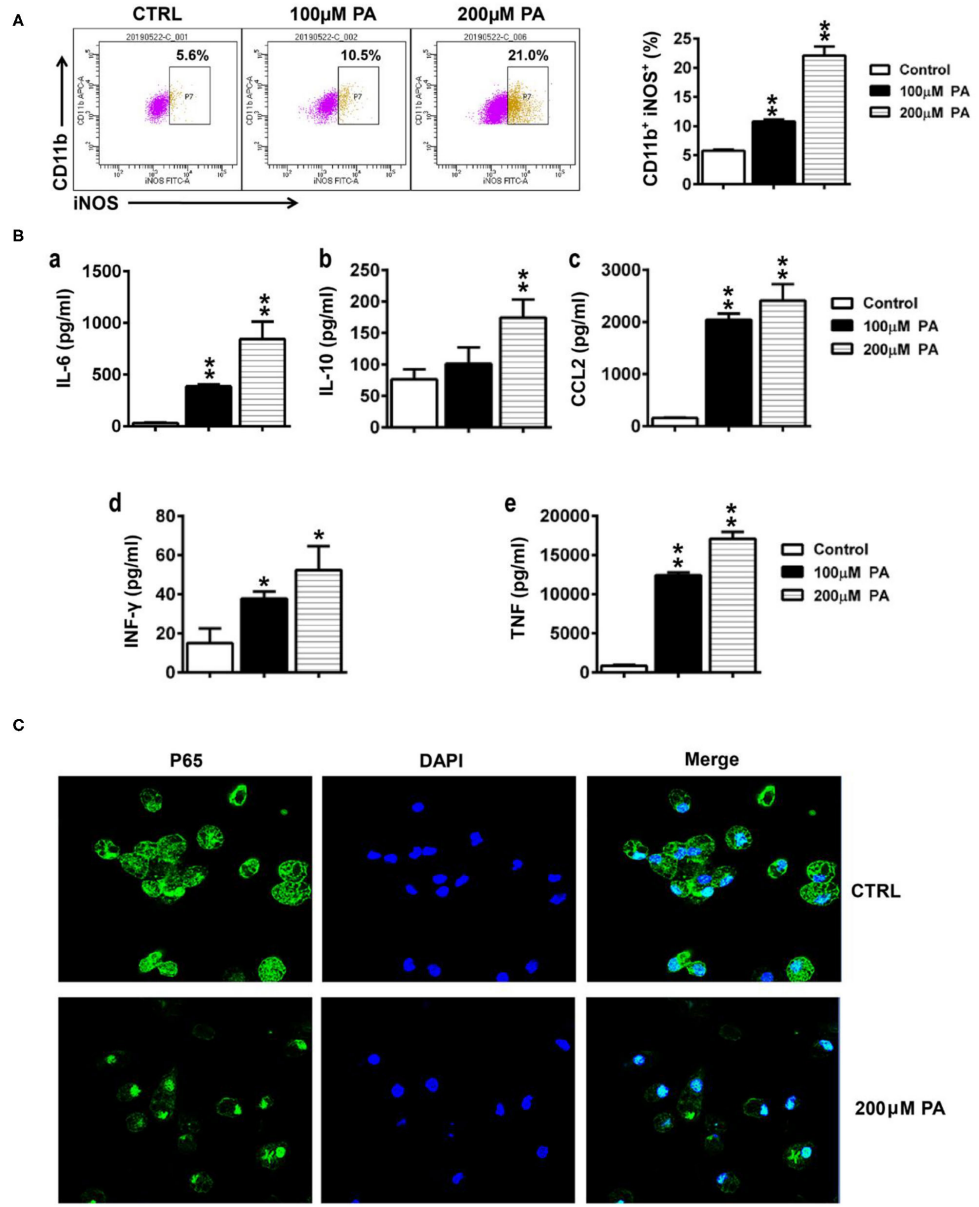
Effect of PA on the secretion of inflammatory factors by macrophages. **(A)** The percentage of iNOS in macrophages was detected by flow cytometry. **(B)** The levels of IL-6, IL-10, CCL2, IFN-γ, and TNF-α in macrophage supernatant were tested using a cytometric bead array. Data is expressed as the mean ± SEM. *P < 0.05, **P < 0.01. **(C)** The localization of P56 in macrophages was assessed by immunofluorescence.

To observe the effect of an HFD on inflammation, we examined the effect of the high-fat metabolite PA on macrophages. As shown in Figure 7A, the percentage of iNOS-positive cells was significantly higher in macrophages after PA (100 or 200 μM) treatment. Moreover, the secretion of inflammatory factors and cytokines, such as IL-6, IL-10, CCL2, IFN-γ, and TNF, significantly increased in the macrophage supernatant after PA (100 or 200 μM) treatment in a dose-dependent manner (Figure 7B). Since P65 is a key transcription factor that regulates inflammatory factors, we investigated the effect of PA on P65 localization in macrophages. As shown in Figure 7C, more P65 protein was observed in macrophage nuclei after PA treatment.

## Discussion

In this study, we found that an HFD can promote the development of DEN-induced serious liver injury and liver cancer. Pathological examination revealed that collagen deposition was lower in the HFD group, whereas inflammatory cell (particularly macrophage) infiltration and inflammatory cytokine levels were higher. *In vitro*, we demonstrated that PA, a high-fat metabolite, can directly activate macrophages and promote inflammatory cytokine secretion. These results suggest that an HFD may promote tumorigenesis by activating macrophages and causing them to release inflammatory cytokines.

After 22 weeks, the survival rates of the DEN, DEN + HF, and control groups were 61.54, 46.15, and 100%, respectively, indicating that the HFD reduced the HCC survival rate. Furthermore, we found that the weight of the DEN-treated mice began to decline after week 16 but there was no significant difference between the DEN + HF and DEN groups during the early stages. However, in the middle and later stages the DEN + HF group lost weight more quickly, which may be related to more rapid tumor progression. Indeed, the number of tumor nodules, liver index, and liver function in the DEN + HF mice were higher than in the DEN group.

γ-GT, PCNA, and cyclin D1 are closely related to HCC cell proliferation. γ-GT is an enzyme synthesized by embryonic hepatocytes that is related to cellular malignancy and HCC occurrence. It is also considered a positive marker of early hepatocyte mutation and a characteristic marker of precancerous lesions. PCNA is a cell proliferation-related protein associated with the metastasis and invasion of HCC (4, 5), whilst cyclin D1 over-expression has been shown to play a key role in the occurrence and development of primary HCC (6). In this study, serum γ-GT, PCNA, and cyclin-1 expression were found to be significantly higher in the DEN + HF group than the DEN group.

We also found that the HFD significantly reduced collagen deposition and increased the number of macrophages infiltrating liver tissues when compared with DEN group. In the tumor microenvironment, collagen can affect tumor cell metabolism, water diffusion, macromolecular transport, gene expression, and angiogenesis by changing the density, direction, and length of crosslinking, among other aspects (7–9). Previously, we found that high protein levels can aggravate collagen deposition and slow tumor development (10). Moreover, there is evidence that continued inflammation can decrease collagen deposition, whilst TNF-α has been shown to reduce the profibrotic collagen-dissolving activity of macrophages and pulmonary fibrosis (11). Therefore, an HFD may aggravate liver inflammation, consistent with the increased NF-κB, IL-1β, and TNF-α expression observed in the DEN + HF group in this study.

Researchers have known for a long time that obesity causes inflammation in the liver and fat tissues (12). An HFD can promote insulin resistance by stimulating chronic inflammation; however, it was shown that when certain immune cells stop producing fatty acids, mice do not develop diabetes or chronic inflammation, even on an HFD (13). Obesity and HFDs can lead to harmful immune system activation and mice fed an HFD have more T cells (14). Studies have found that in addition to fat cells, fat tissue contains many immune cells, including large numbers of macrophages. Fat cells release whole triglycerides in small particles known as adipocyte exosomes (AdExos) which are ingested by macrophages in adipose tissue. Macrophages rapidly degrade the triglycerides in AdExos and release fatty acids (15); thus, macrophages may play an important role in high lipid metabolism. We found that macrophages can be activated by PA to increase iNOS levels and their secretion of IL-6, IL-10, CCL2, INF- γ, and TNF-α. These studies suggest that fatty acids can induce the expression of various inflammatory factors that stimulate macrophages, thus aggravating the inflammatory response.

In our study, we observed that an HFD increased liver macrophages and aggravated inflammation and liver cancer, suggesting that macrophages play an important role in the DEN-induced liver cancer model; however, whether the promotion of liver cancer by an HFD can be reversed in the absence of macrophages is unclear. As demonstrated by previous studies, macrophages are in close interaction with enteric microbiota, which contributes to carcinogenesis and affects treatment outcomes (16, 17). Therefore, both macrophages and intestinal microbiota are considered promising prognositic indicators and valuable targets for new therapeutic approaches. Moreover, recent strong evidence suggests that small intestinal tissue-specific group 2 innate lymphoid cells (ILC2s) are the key to obesity in mice. Mice lacking specific ILCs in the small intestine do not exhibit physiological signs of obesity, such as large amounts of white fat tissue, larger livers, higher blood sugar levels, and impaired insulin resistance (18). This suggests that ILC2s in the small intestine that promote liver fat deposition also may be a primary factor in HFD-associated liver cancer.

In conclusion, HFDs promoted DEN-induced liver cancer, mainly by introducing high-fat metabolites into the liver which activate macrophages and induce inflammation that aggravates HCC.

## Data Availability Statement

The original contributions presented in the study are included in the article/supplementary materials, further inquiries can be directed to the corresponding author/s.

## Ethics Statement

The animal study was reviewed and approved by Ethics Committee of Zhejiang Chinese Medical University.

## Author Contributions

XL, CC, and QS: conceptualization. HF, BT, JL, and MF: methodology. YD: data curation. ZH and LJ: writing—original draft preparation. BC and XL: writing—review and editing. ZH, QS, HF, and XL: funding acquisition. All authors contributed to the article and approved the submitted version.

## Conflict of Interest

The authors declare that the research was conducted in the absence of any commercial or financial relationships that could be construed as a potential conflict of interest.
